# Blood Donation, Being Asian, and a History of Iron Deficiency Are Stronger Predictors of Iron Deficiency than Dietary Patterns in Premenopausal Women

**DOI:** 10.1155/2014/652860

**Published:** 2014-06-11

**Authors:** Kathryn L. Beck, Cathryn A. Conlon, Rozanne Kruger, Anne-Louise M. Heath, Christophe Matthys, Jane Coad, Beatrix Jones, Welma Stonehouse

**Affiliations:** ^1^Institute of Food Nutrition and Human Health, Massey University, Albany, Private Bag 102 904, North Shore City 0745, New Zealand; ^2^Department of Human Nutrition, University of Otago, P.O. Box 56, Dunedin 9054, New Zealand; ^3^Clinical and Experimental Endocrinology, KU Leuven, 3000 Leuven, Belgium; ^4^Institute of Information and Mathematical Sciences, Massey University, Albany, Private Bag 102 904, North Shore City 0745, New Zealand

## Abstract

This study investigated dietary patterns and nondietary determinants of suboptimal iron status (serum ferritin < 20 **μ**g/L) in 375 premenopausal women. Using multiple logistic regression analysis, determinants were blood donation in the past year [OR: 6.00 (95% CI: 2.81, 12.82); *P* < 0.001], being Asian [OR: 4.84 (95% CI: 2.29, 10.20); *P* < 0.001], previous iron deficiency [OR: 2.19 (95% CI: 1.16, 4.13); *P* = 0.016], a “milk and yoghurt” dietary pattern [one SD higher score, OR: 1.44 (95% CI: 1.08, 1.93); *P* = 0.012], and longer duration of menstruation [days, OR: 1.38 (95% CI: 1.12, 1.68); *P* = 0.002]. A one SD change in the factor score above the mean for a “meat and vegetable” dietary pattern reduced the odds of suboptimal iron status by 79.0% [OR: 0.21 (95% CI: 0.08, 0.50); *P* = 0.001] in women with children. Blood donation, Asian ethnicity, and previous iron deficiency were the strongest predictors, substantially increasing the odds of suboptimal iron status. Following a “milk and yoghurt” dietary pattern and a longer duration of menstruation moderately increased the odds of suboptimal iron status, while a “meat and vegetable” dietary pattern reduced the odds of suboptimal iron status in women with children.

## 1. Introduction

Iron deficiency is a major contributor to anemia, which affects nearly one-quarter of the world's population [1]_._ Iron deficiency anemia is associated with poor pregnancy outcome [2], increased susceptibility to infection [3], and decreased work capacity [4]. The health consequences of iron deficiency without anemia are more controversial, but evidence suggests associations with reduced work performance [4] and impaired cognitive function [5]. Iron deficiency is common in both developing and developed countries, such as New Zealand, and premenopausal women are at particular risk. The 2008/09 New Zealand Adult Nutrition Survey found that 12.1% of women aged 31 to 50 years had iron deficiency (serum ferritin (SF) <12 *μ*g/L, zinc protoporphyrin >60 *μ*mol/mol, and hemoglobin (Hb) ≥ 120 g/L) and 6.3% had iron deficiency anemia (SF < 12 *μ*g/L, zinc protoporphyrin >60 *μ*mol/mol, and Hb < 120 g/L) [6]. In addition, many more women are at risk of iron depletion, with a study in New Zealand finding that 23% of women aged 18 to 40 years had a SF that was indicative of low to absent iron stores (i.e., SF < 20 *μ*g/L) [7]. Similarly, the United Kingdom National Diet and Nutrition Survey found that 18 to 30% of women aged 19 to 49 years had a SF < 20 *μ*g/L [8].

Most studies investigating associations between dietary intake and iron status have focused on individual nutrients (e.g., iron) and foods (e.g., meat) [7, 9–16] which has several limitations. People do not eat foods and nutrients alone but as meals consisting of a variety of foods and nutrients that may impact on one another [17, 18]; for example, phytic acid (e.g., from wholegrain breads and cereals) decreases absorption of nonheme iron (e.g., from vegetables) [19]. The analysis of dietary patterns can help to overcome this problem by considering the whole diet and describing how foods are consumed in combination. In recent years, empirically derived dietary patterns have been used to assess the association between dietary intake and anemia [20] and dietary intake and iron status [21, 22]. Previous investigations of the women in our study suggested that those following a “meat and vegetable” dietary pattern had a reduced risk of suboptimal iron status (defined as iron depletion, iron deficiency, or iron deficiency anaemia; SF < 20 *μ*g/L), while women following a “milk and yoghurt” dietary pattern were more likely to have suboptimal iron status [22]. These studies [20–22] have been important for demonstrating the value of dietary patterns as a tool for describing the diet in investigations of iron status.

Nondietary factors also impact on iron status. A New Zealand study of women aged 18 to 40 years found that, in addition to low consumption of animal tissue foods (meat, poultry, and fish), the nondietary factors blood donation, nose bleeds, and menstruation were all associated with mild iron deficiency (SF < 20 *μ*g/L; Hb ≥ 120 g/L) [7]. More recent studies have investigated associations between dietary patterns (rather than individual foods) and iron status, but they have not considered dietary patterns alongside the extensive range of nondietary determinants likely to influence iron status, such as blood loss, so it has been difficult to determine exactly how important dietary patterns are [20–22].

As iron status is affected by both dietary and nondietary determinants, we investigated these in combination to determine the importance of dietary patterns in the context of other risk factors. This study aimed to investigate the relative importance of dietary patterns and nondietary determinants associated with suboptimal iron status (SF < 20 *μ*g/L) in premenopausal women living in Auckland, New Zealand.

## 2. Materials and Methods

The methods are described in detail in Beck et al. [22]. In brief, 404 women aged 18 to 44 years living in Auckland, New Zealand, were recruited for this cross-sectional study. A range of recruitment methods were used including written material (e.g., posters, flyers, email contacts, newspaper articles, and advertisements) and announcements at various events (e.g., lectures, mothers' groups, cooking classes, and sporting events). Exclusion criteria were current pregnancy or breastfeeding, pregnancy in the past year, health problems likely to affect iron status including menorrhagia, and consumption of high dose iron supplements (≥20 mg elemental iron at least 3-4 times per week) in the past three months.

The research was conducted with the understanding and written informed consent of participants. Ethical approval was obtained from the Massey University Human Ethics Committee (Southern A, reference numbers 07/73 and 08/20).

All subjects visited the Human Nutrition Research Unit at Massey University, Auckland, between March and September 2008. At this appointment subjects were interviewed by a researcher, anthropometric measurements were taken, and a validated iron food frequency questionnaire (FFQ) [23] and a validated menstrual blood loss questionnaire [24] were completed.

A venipuncture blood sample was taken for the determination of SF, Hb, and C-reactive protein (CRP) at either the Human Nutrition Research Unit or at Diagnostic MedLab, Auckland. All samples were analysed by Diagnostic MedLab, Auckland, an International Accreditation New Zealand laboratory, with the methods described in Beck et al. [22]. Women were categorised as having sufficient iron stores (SF 20–200 *μ*g/L and Hb ≥ 120 g/L) or suboptimal iron status (SF < 20 *μ*g/L), including iron deficiency anemia (SF < 20 *μ*g/L and Hb < 120 g/L).

During the interview, questions were asked about demographics (e.g., age and ethnicity), lifestyle (e.g., having children, smoking, and use and type of contraception), and medical history (e.g., illness, medication, and supplement use in the past year). Height and weight were measured using the International Society for the Advancement of Kinanthropometry standards [25] and body mass index (BMI) was calculated as weight (kg)/height (m)^2^. As blood loss is known to affect iron status [7], subjects completed a previously validated menstrual blood loss questionnaire (able to differentiate between high and low levels of menstrual blood loss) [24] and questions on blood donation and nose bleeds. The questionnaire was updated to include details on sanitary items (brand and absorbency) that were not available when the questionnaire was developed and was completed online.

The online iron FFQ (described in detail in Beck et al. [23]) was designed to include foods and food groupings containing iron or factors known to affect iron absorption. In a previous study, the iron FFQ was found to be a reproducible and reasonably valid method of assessing frequency of food grouping intake and iron-related dietary patterns [22]. The mean weekly frequency of consumption of each food grouping was determined, and the thirty most frequently consumed food groupings were entered into a factor analysis to determine dietary patterns. Orthogonal varimax rotation was used to assist in the interpretation of factors and seven dietary patterns were identified, explaining 44.3% of the variance in food intake scores. These were “refined carbohydrate and fat,” “Asian,” “healthy snacks,” “meat and vegetables,” “high tea and coffee,” “bread and crackers,” and “milk and yoghurt” dietary patterns. These patterns are explained further in Beck et al. [22].

All statistical analyses were performed using Predictive Analytics Software (PASW) statistics 18 (SPSS Inc., Chicago, IL, USA). Two-sided tests were used for all analyses and a *P* value of <0.05 was considered statistically significant.

Descriptive statistics including the mean (standard deviation) and frequency summary statistics were used to describe the study population. Comparisons between groups were made using the independent *t*-test for continuous variables and chi-square test to investigate categorical variables.

Simple logistic regression analysis was used to investigate potential determinants likely to be associated with suboptimal iron status. These determinants included age, BMI, ethnicity (European, Asian, or other), being born in New Zealand, having children, blood donation in the past year, having nose bleeds, estimated menstrual blood loss, duration of menstrual periods, using oral contraception, using an intrauterine device, being a user of low dose iron supplements or multivitamins in the past year, previous iron deficiency (defined as having iron depletion, iron deficiency, or iron deficiency anemia in the past), smoking, and all seven dietary patterns obtained from the iron FFQ [22]. Age, BMI, estimated menstrual blood loss, duration of menstrual periods, and dietary patterns (factor scores) were entered into the model as continuous variables. All other variables were treated as binary variables. Reference categories were the absence of the determinant and being of European descent (for ethnicity).

Variables with a univariate *P* value <0.20 were entered into the multiple logistic regression analysis. This value was chosen as univariate *P* values ≥0.20 were considered unlikely to contribute to a model containing other potential determinants of suboptimal iron status. As estimated MBL was calculated using duration of menstrual periods; both variables could not be included in the multiple logistic regression analysis. Duration of menstrual period was entered into the model as it had a higher level of significance following simple logistic regression analysis (*P* = 0.006 versus *P* = 0.14) and has been used more often within the literature [7, 12, 26, 27]. Multicollinearity existed between age and having children (*r* = 0.75 (*P* < 0.001)); however both variables were included in the model selection procedure as both could potentially contribute to suboptimal iron status.

Forward stepwise multiple logistic regression with the entry criterion set at *P* < 0.05 was used to determine which variables to include in the final multivariate model. Residual statistics (namely, the standardised residual, Cook's distance, leverage, and DFBeta for the constant and determinants) were examined to ensure no cases exerted an undue influence on the final logistic regression model. After the main effects were determined by the stepwise procedure, two-way interactions between binary and continuous variables that passed the initial screen (e.g., blood donation versus having children and “meat and vegetable” dietary pattern versus blood donation) were added individually to the main effects model to test their significance.

## 3. Results

Of the 404 women recruited, a total of 375 were included in the final analysis (twenty-nine were excluded for the following reasons: elevated iron stores (SF >200 *μ*g/L) (*n* = 2), anemia without iron deficiency (SF ≥ 20 *μ*g/L, Hb < 120 g/L) (*n* = 16), CRP >10 mg/L (as SF is an acute phase protein and is increased during inflammation and infection [28]) (*n* = 4), and unavailable dietary data (*n* = 4) or blood results (*n* = 3)). Of those eligible, 305 (81.3%) had sufficient iron stores, and seventy (18.7%) had suboptimal iron status, of whom twenty (5.3%) had iron deficiency anemia.

Table 1 shows the subjects' characteristics. Women with suboptimal iron status were more likely to be older (*P* = 0.02), to be of Asian ethnicity (*P* = 0.002), to have children (*P* = 0.002), to have donated blood in the past year (*P* < 0.001), to have a longer duration of menstrual periods (*P* = 0.004), and to have had iron deficiency previously (*P* = 0.01) and were less likely to use oral contraception (*P* = 0.013), compared with women who had sufficient iron stores.

Eleven variables with a *P* value of <0.20 on univariate analysis were entered into the forward stepwise multiple logistic regression analysis: age, ethnicity (European versus Asian), having children, blood donation in the past year, duration of menstrual period, using oral contraception, previous iron deficiency, and the following dietary patterns: “meat and vegetable,” “milk and yoghurt,” “bread and crackers,” and “refined carbohydrate and fat.” Interactions between Asian ethnicity and the “bread and crackers” dietary pattern, between blood donation and the “meat and vegetable” dietary pattern, between blood donation and the “bread and crackers” dietary pattern, and between having children and the “meat and vegetable” dietary pattern also passed the initial screen. These interactions were also entered into the multiple logistic regression analysis. The following variables did not meet the screening criterion so they were not included in the multiple logistic regression analysis: BMI, ethnicity (European versus other), being born in New Zealand, having nose bleeds, estimated MBL, using intrauterine device, using low dose iron supplements or multivitamins in the past year, smoking, and the “Asian,” “healthy,” and “high tea and coffee” dietary patterns.

Within the final multiple logistic regression model, a one standard deviation (SD) change in the factor score above the mean for the “milk and yoghurt” dietary pattern increased the odds of having suboptimal iron status by 1.44 times (44.0%) (*P* = 0.012) (see Table 2). The “milk and yoghurt” dietary pattern explained only 1.9% of the variance in suboptimal iron status (increase in the Hosmer and Lemeshow *R* ^2^), while the remaining determinants explained 20.2% of the variance in suboptimal iron status. Donation of blood in the past year compared with no blood donation in the past year increased the odds of suboptimal iron status by six times (*P* < 0.001), while being of Asian ethnicity compared to European ethnicity increased the odds of suboptimal iron status by almost five times (*P* < 0.001). Having previously had iron deficiency increased the odds of suboptimal iron status by almost three times compared with not previously having iron deficiency (*P* = 0.016), and a longer duration of menstrual periods increased the odds of suboptimal iron status by 1.4 times (for every additional day of menstruation in each cycle) (*P* = 0.002). A significant interaction effect between having children and the “meat and vegetable” dietary pattern was also observed in the final model (*P* < 0.001).

An analysis stratified on “having children” was done to understand how scores on the “meat and vegetable” dietary pattern affected women with and without children. Women in both groups had a similar range of dietary pattern scores. After adjusting for other variables, a one SD change in the factor score above the mean for a “meat and vegetable” dietary pattern reduced the odds of having suboptimal iron status by 0.21 times (79.0%) (*P* = 0.001) in women who had children. In women who did not have children, following a “meat and vegetable” dietary pattern was not a determinant of suboptimal iron status (*P* > 0.05) (see Table 3).

## 4. Discussion

To our knowledge, this is the first study to investigate the role of dietary patterns as well as nondietary factors simultaneously as determinants of suboptimal iron status in premenopausal women. Donation of blood in the past year, being of Asian ethnicity, and having had iron deficiency in the past were the strongest predictors, substantially increasing the odds of having suboptimal iron status. A change of one SD in the factor score above the mean for the “milk and yoghurt” dietary pattern had a similar predictability on the odds ratios of suboptimal iron status as an additional day of menstrual blood loss. Following a “meat and vegetable” dietary pattern resulted in a statistically significant decrease in the odds of suboptimal iron status only in women who had children.

Blood donation in the past year was the strongest determinant of suboptimal iron status, increasing the odds of suboptimal iron status by six times as an individual moved from a nonblood donor to a blood donor state. This finding is in agreement with several other studies which have found blood donation to be associated with a lowered iron status in women [7, 9, 12, 15, 16]. Approximately 1–1.35 mg of iron per day is lost from the body each time blood is donated assuming six months between blood donations [29]. However, the effect of blood donation on iron status may be due to a transient effect in the first few months following blood donation [7]. Less frequent donation of blood (currently in New Zealand, individuals can donate blood up to four times per year [30]) or discouragement of blood donation in women at risk of iron deficiency may help to reduce their risk of developing suboptimal iron status.

Being of Asian ethnicity increased the odds of suboptimal iron status by almost five times compared with being of European ethnicity. This group of Asian women comprised predominantly Chinese (41.8%) and Indian (29.1%) women. Most studies in premenopausal women have not investigated associations between ethnicity and iron status, possibly due to less ethnically diverse study populations. The reasons for a higher prevalence of suboptimal iron status in Asian women are not known but may be due to genetic [31] (e.g., hemoglobinopathy) or lifestyle (e.g., dietary and socioeconomic) factors. More research is needed to determine why Asian women appear to have an increased likelihood of suboptimal iron status. Furthermore, there may be a component within the diet of Asian women which was not captured by the dietary patterns in this study. Another New Zealand study found Māori, Pacific Island, and Asian female high school students to have two to four times the odds of iron deficiency and iron deficiency anemia compared with European high school students [32]. In the United States (NHANES III), Mexican American women were more likely to have iron deficiency anemia than non-Hispanic white women after adjustment for iron supplement use, parity, and poverty level [33]. In the current study, being of “other” ethnicities (which included women of Māori and Pacific Island descent) was not found to be significantly associated with suboptimal iron status. While we had good power for detecting large differences in iron status between women of different ethnicities, it is possible that small to moderate differences in iron status between European and “other” ethnic groups may have been detected with a larger sample size.

Previous iron deficiency was found to be more than double the odds of suboptimal iron status in this study compared with not having iron deficiency previously. Heath et al. [7] found no significant differences in the rates of previous low iron status between women with mild iron deficiency and those without. Other studies in premenopausal women have not investigated associations between iron status and previous iron deficiency.

The “milk and yoghurt” dietary pattern remained a significant determinant of suboptimal iron status after controlling for dietary and nondietary determinants. However, the “milk and yoghurt” dietary pattern only explained 1.9% of the variance in suboptimal iron status. A movement of one SD above the mean on the “milk and yoghurt” dietary pattern increased the odds of suboptimal iron status by 44.0% (odds ratio (OR) 1.44). Based on subjects whose factor scores were closest to the mean and one SD above the mean for the “milk and yoghurt” dietary pattern, this was equivalent to an increase in consumption of milk in drinks from 1.0 (mean) to 2.5 (1 SD above the mean) times per week and milk in food from 5.0 to 7.0 times per week (yoghurt intake was not different in these individuals). Calcium (found in high quantities in milk and yoghurt) inhibits heme and nonheme iron absorption [34].

A significant interaction was observed between having children and the “meat and vegetable” dietary pattern. After controlling for other possible determinants of iron status, a one SD increase above the mean on the “meat and vegetable” dietary pattern reduced the odds of suboptimal iron status by 79.0% (OR 0.21) in women who had children. There was not a statistically significant association in the women without children. Based on subjects whose factor scores for the “meat and vegetable” dietary pattern were closest to the mean and one SD above the mean, this equalled an increase in consumption of chicken from 1.0 (mean) to 2.5 (1 SD above the mean) times per week, broccoli and carrots (both 2.5 to 5.0 times per week), and lettuce (7.0 to 17.5 times per week). Beef and capsicum intake were not different in these individuals. Muscle tissue (meat) is a well-known enhancer of nonheme iron absorption [35] and a number of cross-sectional studies have found meat to be associated with improved iron status [7, 13, 16]. Capsicum and broccoli contain ascorbic acid, a well-known enhancer of iron absorption [36]. The literature is conflicting regarding associations between having children and iron status. Looker et al. [37] found an association between increased parity and iron deficiency, while other studies have found no association between having children [7] or number of children [31] and iron status. It is not clear why the significant association between having children and the “meat and vegetable” dietary pattern was only observed in women with children. Collinearity existed in our dataset between age and having children, and the difference between the children and no children groups may also reflect the effect of childbearing age (although age was controlled for in the model).

A longer duration of bleeding at each menstrual period was associated with suboptimal iron status. Heath et al. [7] found a greater duration and extent of menstrual bleeding to be associated with mild iron deficiency (SF < 20 *μ*g/L, Hb ≥ 120 g/L). Most [12, 26, 27] but not all studies [31] have shown a greater duration of menstrual period to be inversely associated with iron status. A reduced iron status has been found to be associated with both self-reported heavy menstrual blood loss [14, 31] and increased menstrual blood loss determined by direct measurement [10]. Asakura et al. [14] found a decreased likelihood of iron deficiency in women with irregular or no menstrual cycles.

Oral contraceptive agents are known to decrease menstrual blood loss and have been found to be positively associated with SF concentrations [13], although other studies have found no significant associations with iron status [12, 16, 26]. In the present study, women with sufficient iron stores were more likely to use oral contraceptives than women with suboptimal iron status. However, when considered alongside other potential determinants of iron status, their use did not significantly affect the odds of suboptimal iron status. These findings are in agreement with Heath et al. [7] who found that once the extent and duration of menstrual bleeding were controlled for, there was no effect of oral contraceptive use on risk of mild iron deficiency.

As far as we are aware, this is the first study to investigate possible associations between dietary patterns and a wide range of nondietary determinants (including blood loss and ethnicity) and iron status in premenopausal women. Unlike previous studies investigating associations between iron status and dietary patterns [20, 21], we used an FFQ validated specifically for dietary patterns [23], and iron status was determined using both SF (a measure of iron stores) and Hb (to indicate anemia) [38]. CRP was also measured to ensure SF was not affected by infection [28]. However, as this study relied on a relatively small convenience sample of women, caution must be used when applying the results to all premenopausal women. Furthermore, selection bias may have influenced the results of the study and the cross-sectional study design meant that causality could not be determined. It is also difficult to compare the results of this study with earlier studies due to inconsistencies in study design including the indices and cut-off points used to define iron deficiency, the populations studied, and the variables investigated.

## 5. Conclusions

In this population of premenopausal women, donation of blood, being of Asian ethnicity, and having had iron deficiency in the past were all stronger predictors of suboptimal iron status than differences in dietary patterns. A one SD increase in the factor score for the “milk and yoghurt” dietary pattern and an additional day of menstruation in each cycle both increased the odds of suboptimal iron status moderately, while a “meat and vegetable” dietary pattern reduced the odds of suboptimal iron status, but only in women who had children. This study has shown that both dietary patterns and nondietary determinants are associated with suboptimal iron status in premenopausal women and should be considered when identifying and treating women at risk of suboptimal iron status.

## Figures and Tables

**Table 1:** Characteristics of study subjects with and without suboptimal iron status.

Characteristic	Subjects with sufficient iron stores (*n* = 305)^a ^	Subjects with suboptimal iron status (*n* = 70)^b ^	*P* value^c^
Age (year)	27.5 ± 8.4	30.1 ± 9.0	0.02
BMI (kg/m^2^)	23.5 ± 4.1	23.0 ± 3.2	0.35
SF (*μ*g/L)	52.9 ± 27.0	13.0 ± 4.3	<0.001
Hb (g/L)	133.7 ± 8.0	124.9 ± 10.5	<0.001
Ethnicity			
European	239 (78.6)	43 (61.4)	0.002
Asian	36 (11.8)	20 (28.6)	
Other	29 (9.5)	7 (10.0)	
Born in New Zealand	177 (58.2)	35 (50.0)	0.23
Having children	80 (26.3)	32 (45.7)	0.002
Any blood donation in the past year	24 (7.9)	19 (27.1)	<0.001
Having nose bleeds	30 (9.9)	8 (11.4)	0.83
Estimated menstrual blood loss (blood loss units)	39.0 ± 31.5	45.8 ± 32.8	0.137
Duration of menstrual period (d)	4.9 ± 1.4	5.4 ± 1.4	0.004
Using oral contraception	99 (32.7)	12 (17.1)	0.013
Using intrauterine device^d^	17 (5.6)	2 (2.9)	0.547
Used low dose iron supplements or multivitamins in the past year	87 (28.7)	21 (30.0)	0.88
Previous iron deficiency	116 (38.2)	39 (55.7)	0.01
Smoker^d^	21 (6.9)	3 (4.3)	0.591

Data are mean ± standard deviation or *n* (%).

^a^SF ≥ 20 *μ*g/L and Hb ≥ 120 g/L.

^b^SF < 20 *μ*g/L (Hb < 120 or ≥120 g/L).

^c^ *P* value is for a test of differences between groups as assessed by the independent *t*-test (continuous data) or chi-square test (categorical data) (adequate expected values).

^d^Difference between groups assessed using Fisher's exact test as expected values <5.0.

BMI: body mass index; Hb: hemoglobin; SF: serum ferritin.

**Table 2:** Results of stepwise multiple logistic regression identifying determinants of suboptimal iron status.

Variable	OR^a^	95.0% CI for OR	*P* value
Any blood donation in the past year	6.00	2.81, 12.82	<0.001
Asian ethnicity	4.84	2.29, 10.20	<0.001
Previous iron deficiency	2.19	1.16, 4.13	0.016
Milk and yoghurt dietary pattern (SD)	1.44	1.08, 1.93	0.012
Duration of menstrual period (d)	1.38	1.12, 1.68	0.002
Meat and vegetable dietary pattern (SD) × having children	0.17	0.08, 0.39	<0.001

^a^Change in odds of suboptimal iron status occurring for each unit change in determinant variable. If >1.0, as determinant variable increases, odds of suboptimal iron status increase. If <1.0, as determinant variable increases, odds of suboptimal iron status decrease.

*R* ^2^ = 0.22 (Hosmer and Lemeshow), 0.19 (Cox and Snell), and 0.31 (Nagelkerke). Model *χ* ^2^ = 79.32.

CI: confidence interval; d: days; OR: odds ratio; SD: standard deviation.

**Table 3:** Following a “meat and vegetable” dietary pattern and odds of suboptimal iron status in women with children and women without children.

	Variable	OR^a^	95.0% CI for OR	*P* value
Women with children (*n* = 112)	Meat and vegetable dietary pattern (SD)	0.21	0.08, 0.50	0.001
Women without children (*n* = 262)	Meat and vegetable dietary pattern (SD)	0.75	0.49, 1.14	0.175

^a^Adjusted for blood donation in the past year, Asian ethnicity, previous iron deficiency, “milk and yoghurt” dietary pattern, and duration of menstrual period using forced entry multiple logistic regression analysis.

CI: confidence interval; OR: odds ratio; SD: standard deviation.
